# Activity of lapatinib a novel HER2 and EGFR dual kinase inhibitor in human endometrial cancer cells

**DOI:** 10.1038/sj.bjc.6604278

**Published:** 2008-03-11

**Authors:** G E Konecny, N Venkatesan, G Yang, J Dering, C Ginther, R Finn, M Rahmeh, M Schoenberg Fejzo, D Toft, S-W Jiang, D J Slamon, K C Podratz

**Affiliations:** 1 Division of Gynecologic Surgery, Department of Obstetrics and Gynecology, Mayo Clinic, Rochester, MN, USA; 2 Division of Hematology–Oncology, Department of Medicine, David Geffen School of Medicine, Jonsson Comprehensive Cancer Center, University of California (UCLA), Los Angeles, CA, USA; 3 Department of Obstetrics and Gynecology, Klinikum Grosshadern, Ludwig Maximilians Universität München, Munich, Germany

**Keywords:** HER2/neu, EGFR, endometrial cancer, lapatinib

## Abstract

In this study, we explore the therapeutic potential of lapatinib a selective inhibitor of both the EGFR and HER2 tyrosine kinases for the treatment of endometrial cancer. The effect of lapatinib on tumour cell growth and receptor activation was studied in a panel of human endometrial cancer cell lines. Candidate molecular markers predicting sensitivity were assessed by baseline gene expression profiling, ELISA, and western blot analyses. Multiple drug effect/combination index (CI) isobologram analysis was used to study the interactions between chemotherapeutic drugs and lapatinib. Concentration-dependent anti-proliferative effects of lapatinib were seen in all endometrial cancer cell lines tested, but varied significantly between individual cell lines (IC_50_ range: 0.052–10.9 *μ*mol). HER2 overexpression or increased expression of EGFR was significantly associated with *in vitro* sensitivity (*P*=0.024 or 0.011, respectively). Lapatinib exerts growth inhibition in a PTEN-independent manner. Sensitive cell lines also exhibited increased expression of EGFR ligands or HER3. In contrast, lapatinib-resistant cell lines exhibited high androgen receptor (AR) levels or epithelial-to-mesenchymal transition (post-EMT) features. In endometrial cancer cells, at a wide range of clinically achievable drug concentrations, additive and synergistic interactions were observed for lapatinib plus carboplatin, paclitaxel, docetaxel, and doxorubicin. These observations provide a clear biologic rational to test lapatinib as a single agent or in combination with chemotherapy in endometrial cancer with HER2 overexpression. Expression of EGFR, its ligands, HER3, AR, and post-EMT markers warrant further evaluation to help define patients with HER2-nonoverexpressing endometrial cancer most likely to benefit from lapatinib.

Endometrial cancer is the most common malignancy of the female reproductive tract. Despite recent advances in the molecular characterisation of endometrial cancer, current treatment strategies do not include target-based therapies (Murphy, 1994; Mariani *et al*, 2005). Among the peptide growth factor receptors frequently implicated in endometrial cancer are members of the type I receptor tyrosine kinase family, which includes HER1 (epidermal growth factor receptor (EGFR)), HER2, HER3, and HER4 (Srinivasan *et al*, 1999; Santin *et al*, 2002). Amplification of the HER2 gene, resulting in overexpression of the receptor, is more commonly found in nonendometrioid endometrial cancers (type II endometrial cancers) and is associated with an aggressive form of the disease with significantly shortened disease-free survival and overall survival (Hetzel *et al*, 1992; Acharya *et al*, 2005). Recent studies including patients with endometrial cancer of diverse histologic type demonstrated HER2 gene amplification in 17/58 (29%) or 11/26 (42%) of uterine serous papillary cancers and 8/63 (13%) grade 3 endometrioid endometrial cancers (Santin *et al*, 2005; Morrison *et al*, 2006).

Epidermal growth factor receptor expression has been demonstrated in 43–67% of patients with endometrial cancer and has similarly been associated with significantly shortened disease-free and overall survival (Khalifa *et al*, 1994; Scambia *et al*, 1994; Niikura *et al*, 1995). Moreover, endometrial cancer demonstrated significantly higher expression levels of the EGFR ligands TGF-*α* and amphiregulin compared with normal endometrium (Pfeiffer *et al*, 1997; Ejskjaer *et al*, 2007). The central role of HER2 and EGFR in growth and differentiation of both normal and malignant cancer cells and their availability to extracellular manipulation make both HER2 and EGFR attractive targets for pharmacological intervention. Recently, agents have been developed that simultaneously inhibit both the EGFR and HER2 epithelial growth factor receptors (Rusnak *et al*, 2001a). Lapatinib is such a synthetic small molecule inhibitor of the HER2 and EGFR tyrosine kinases (Rusnak *et al*, 2001b). Inhibiting both receptors may be particularly attractive as interactions between HER2 and EGFR provide a mechanism for signal diversification and augmentation (Konecny *et al*, 2006).

Our primary aim was to explore the *in vitro* effects of this dual receptor tyrosine kinase inhibitor (TKI) in an unbiased way using a large panel of 19 endometrial cancer lines that express variable levels of HER2 and EGFR. Subsequently, all cell lines were molecularly characterised using Agilent Microarrays. Although this technology allows us to examine thousands of genes simultaneously the identification of a more limited number of markers that predict response to lapatinib in endometrial cancer cells might be more useful for independent validation in clinical studies. Importantly, for this study markers were selected on the basis of their potential biologic relevance in HER2 and EGFR signalling. Therefore, we correlated the IC_50_ values with the relative expression levels of EGFR, HER2, HER3, and HER4 as well as the ligands of EGFR such as TGF-*α*, EGF, amphiregulin, HB-EGF, betacellulin, epiregulin, neuregulin 1, and neuregulin 2. Next, we correlated the relative expression of the steroid hormone receptors oestrogen receptor-*α* (ER-*α*) or -*β* (ER-*β*), progesterone receptor (PR), and androgen receptor (AR) with *in vitro* response to lapatinib, because recent studies suggest that EGFR inhibitors may be particularly active in a subset of breast tumours described as ‘triple negative’ (ie, negative ER, PR, and HER2 expression) (Finn *et al*, 2006). Furthermore, the identification of novel therapies in particularly aggressive tumour cells that have undergone a shift from epithelial differentiation towards a mesenchymal phenotype is a clinical priority. This process referred to as epithelial-to-mesenchymal transition (EMT), increases motility and invasiveness of tumour cells and is often considered a prerequisite for tumour infiltration and metastasis (Thiery and Sleeman, 2006). To further determine the potential clinical role of lapatinib in particularly aggressive endometrial cancer cells we compared the *in vitro* effects of lapatinib between both of these subtypes.

Finally, we used multiple drug effect/combination index (CI) isobologram analysis to study the efficacy of chemotherapeutic drugs plus lapatinib combinations tested against lapatinib-sensitive HER2-amplified/overexpressing or EGFR-expressing endometrial cancer cells.

In summary, the current studies were intended to provide a rational to test lapatinib as a single agent or in combination with chemotherapy in patients with high-risk primary or metastatic endometrial cancer and to identify candidate markers that may help define subsets of patients most likely to benefit from treatment with lapatinib.

## MATERIALS AND METHODS

### Cell lines, cell culture, and reagents

The effects of lapatinib on malignant cell growth were studied in a panel of 19 established human endometrial cancer cell lines. The established human endometrial carcinoma cell lines KLE, RL-95-2, AN3CA, HEC1A, and HEC1B were obtained from American Type Culture Collection (Rockville, MD, USA). The established human endometrial cancer cell lines MFE280, MFE296, MFE319, EFE184, and EN were obtained from the German Tissue Repository DSMZ (Braunschweig, Germany). Ishikawa cells were obtained from the European Collection of Cell Cultures (ECACC, Salibury, Wiltshire, England). The established human endometrial carcinoma cell lines HEC155, SNG-II, and SNG-M were obtained from the Japanese Health Science Research Resources Bank (Osaka, Japan). The cell lines SPAC1S and SPAC1L were provided by the laboratory of Dr Y Hirai from the Department of Gynecology, Cancer Institute Hospital (Tokyo, Japan). The cell line EN1 was provided by Dr V Möbus from Department of Gynecology at the University of Ulm (Germany). USPC1 and USPC2 cells were provided by Dr A Santin from the Department of Obstetrics and Gynecology, Division of Gynecologic Oncology at the University of Arkansas (Little Rock, AR, USA). USPC2, RL-95-2, SPAC1L, SPAC1S, and HEC1B cells were cultured in RPMI medium 1640 supplemented with 10% heat-inactivated fetal bovine serum, 2 mM glutamine, and PSF (Irvine Scientific, Santa Ana, CA, USA). HEC1A cells were cultured in McCoy's medium supplemented with 10% heat-inactivated fetal bovine serum and PSF (Irvine Scientific). SNG-II and SNG-M cells were cultured in Ham's F-12 supplemented with 10% heat-inactivated fetal bovine serum and PSF (Irvine Scientific). The remaining cell lines were cultured in DMEM medium supplemented with 10% heat-inactivated fetal bovine serum, 2 mM glutamine, and PSF (Irvine Scientific). Lapatinib was provided by GlaxoSmithKline (Research Triangle Park, NC, USA) as a 10-mM concentrated stock solution in dimethyl sulphoxide.

### Quantitation of HER2 and EGFR expression

HER2 and EGFR protein content in all cell lines was measured by ELISA as described previously (Konecny *et al*, 2006) (HER2 ELISA; Oncogene Research Products, Calbiochem, San Diego, CA, USA and EGFR ELISA; R&D Systems, Minneapolis, MN, USA).

### Proliferation assays

Cells were plated into 24-well plates at a density of 2 to 5 × 10^5^ and grown in cell line-specific media without or with increasing concentrations of lapatinib (ranging between 0.031 and 10 *μ*M). Cells were harvested by trypsinisation on day 7 and counted using a particle counter (Z1; Beckman Coulter Inc., Fullerton, CA, USA). Growth inhibition was calculated as a percentage of the untreated controls. Experiments were performed three times in duplicate for each cell line. The log of the fractional growth inhibition was then plotted against the log of the drug concentration, and the IC_50_ values were interpolated from the resulting linear regression curve fit (CalcuSyn; Biosoft, Ferguson, MO, USA).

### Western blots and immunoprecipitation

Cells in log-phase growth were washed in PBS and lysed at 4°C in lysis buffer. Insoluble material was cleared by centrifugation at 10 000 **g** for 10 min. Protein was quantitated using BCA (Pierce Biochemicals, Rockford, IL, USA), resolved by SDS-PAGE, and transferred to nitrocellulose membranes (Invitrogen, Carlsbad, CA, USA). HER2, EGFR, and PTEN expression were detected, respectively, by monoclonal anti-HER2 (Ab-3; Calbiochem), anti-EGFR antibodies (Pharmingen; San Diego, CA, USA), and anti-PTEN antibodies (PTEN 7974; Santa Cruz Biotechnology, Santa Cruz, CA, USA). For the evaluation of concentration-dependent activity of lapatinib on HER2, EGFR, AKT, and ERK Phosphorylation, USPC1, USPC2, RL-95-2, HEC155, and SNG-II cells in log-phase growth were treated with increasing doses of lapatinib (0.5–5 *μ*M) for 1 h prior to cell lysis. Tyrosine phosphorylation of HER2 and EGFR was analysed as follows. Immunoprecipitations were performed by allowing 250 *μ*g protein lysate to incubate with 3 *μ*g monoclonal anti-HER2 (Ab-3) or anti-EGFR antibody (Ab-1; Calbiochem), and Protein A/G-agarose (Santa Cruz Biotechnology) at 4°C overnight with gentle agitation. The immunoprecipitates were washed three times in lysis buffer and then denatured in Lemmli's buffer prior to SDS-PAGE. Immunoblotting was performed using a monoclonal anti-phosphotyrosine antibody (Upstate, Charlottsville, VA, USA). Detection was performed using ECL Plus chemifluorescent reagent (Amersham Biosciences, Piscataway, NJ, USA) and imaging of the resulting western blots was performed using the chemifluorescence method by Typhoon 9400 (Amersham Biosciences). Phospho-AKT and phospho-ERK were detected by polyclonal anti-pAKT (Ser473) and anti-pERK (Thr202/Thr204) antibodies (Cell Signaling, Beverly, MA, USA).

### Fluorescence *in situ* hybridisation

HER2 gene copy number was analysed using fluorescence *in situ* hybridisation (FISH). In 12 established endometrial cancer cell lines (EN, Ishikawa, KLE, RL-95-2, HEC155, HEC1A, HEC1B, MFE319, MFE280, MFE296, USPC1, and USPC2). The cell lines were treated with Colcemid (0.05 g ml^−1^) for 2–4 h to obtain metaphase preparations. All samples were fixed in methanol/acetic acid (3 : 1). Specimen preparation, hybridisation, and microscopy were performed as previously described (Pauletti *et al*, 2000). A multicolor topo-II-alpha spectrum orange, HER2 spectrum green, and CEP17 spectrum aqua probe was used (Vysis Inc., Downers Grove, IL, USA). Data were expressed in terms of HER2/neu and topo-II-alpha genes per chr.17cen., with two HER2 or topo-II-alpha genes per chr.17cen. as the biologic cutoff point to distinguish cell lines with or without HER2 and topo-II-alpha amplification.

### Multiple drug effect analysis

Aliquots of 3 to 5 × 10^3^ USPC1, USPC2, RL-95-2, and HEC155 cells were plated in 96-well microdilution plates. Following cell adherence (24 h), experimental media containing either control media, lapatinib, chemotherapeutic agent, or the combination (lapatinib plus chemotherapeutic agent) were added to appropriate wells in duplicate, and serial two-fold dilutions were performed to span clinically relevant concentration ranges for the dose–effect analysis for lapatinib and drug combinations. Following incubation for 72–120 h, plates were washed with PBS and stained with 0.5% *N*-hexamethylpararosaniline (crystal violet) in methanol. Sorenson's buffer (0.025 M sodium citrate, 0.025 M citric acid in 50% ethanol) (0.1 ml) was added to each well, and the plates were analysed in an ELISA plate reader at 540 nm wavelength. For each assay, the log of the fractional growth inhibition was plotted against the log of the drug concentration and the linear regression curve fit correlation coefficient (*r*-value) was calculated. Multiple drug effect analysis was performed using computer software (Biosoft) as described in detail (Pegram *et al*, 2004). In this analysis, synergy is defined as CI values significantly lower than 1.0, antagonism as CI values significantly higher than 1.0, and additivity as CI values equal to 1.0.

### Microarray analysis

Agilent microarray analyses were developed for each cell line. Briefly, cells were grown to log phase, and then RNA was extracted using the RNeasy Kit (Qiagen, Valencia, CA, USA). The purified RNA was eluted in 30–60 *μ*l DEPC water and the quantity of RNA measured by spectral analysis using the Nanodrop Spectrophotometer. RNA quality was determined by separation of the RNA via capillary electrophoresis using the Agilent 2000 Bioanalyzer. Microarrays of endometrial cancer cell lines were then performed on the Agilent Human 1A V1 chip. Characterisation of individual breast cancer cell lines by comparison to an endometrial cancer cell line mixed reference pool was conducted on a single slide in which the mixed pool RNA was labelled with cyanine-3 and the individual cell lines with cyanine-5. The mixed reference pool consisted of equal amounts of cRNA from endometrial cancer cell lines that were selected to be representative of a range of the various known endometrial cancer subtypes. The reference includes 18 cell lines (all mentioned above except USPC1). Microarray slides were read using an Agilent Scanner, and the Agilent Feature Extraction software version 7.5 was used to calculate gene expression values. Extracted data were imported into Rosetta Resolver 5.1 to create expression profiles for each individual endometrial cell line experiment. Cluster analysis was performed in Resolver and cell line profiles were exported to Excel (Microsoft, Redmond, WA, USA) for additional analysis.

### Statistical methods

Associations between the expression levels of biomarkers and the IC_50_ values were analysed using Spearman's *ρ* correlation, and differences in the IC_50_ values between subgroups compared using the Mann–Whitney *U*-test. All statistical tests were two sided. The threshold for upregulation was set to log (ratio) >0.13 with a *P*-value <0.01, and downregulation was defined as log (ratio) ⩽0.13 with a *P*-value <0.01. The *P*-values were determined according to the Agilent error model when the feature-extracted data were imported into Resolver. This study was reviewed and approved by the Mayo Clinic Foundation Institutional Review Board.

## RESULTS

### Activity of lapatinib in human endometrial cancer cells

**Figure 1:**
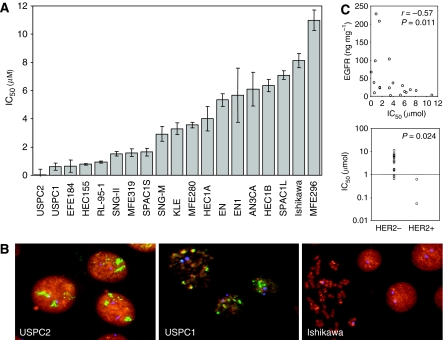
(**A**) Growth inhibitory effects of lapatinib were studied across a panel of endometrial cancer cell lines. Cells were grown in cell line-specific media without or with increasing doses of lapatinib (ranging between 0.031 and 10 *μ*M). Cells were trypsinised and counted after 7 days of treatment. The percentage of inhibition was calculated compared with untreated controls. The log of the fractional growth inhibition was plotted against the log of the drug concentration. The dose achieving 50% growth inhibition (IC_50_) was interpolated from the resulting linear regression curve fit. Cell lines are ordered from left to right from low to high IC_50_ values. Error bars indicate the SE of the mean value. Mean is derived from three replicate experiments. (**B**) HER2 and topo-II-*α* gene copy number were analysed by FISH using a multicolor topo-II-alpha spectrum orange, HER2 spectrum green, and CEP17 spectrum aqua probe (Vysis Inc.). (**C**) Epidermal growth factor receptor and HER2 expression were assessed by ELISA for each of the endometrial cancer cell lines. Epidermal growth factor receptor was correlated with the corresponding IC_50_ value (Spearman's *ρ* correlation coefficient). For HER2, the IC_50_ values were compared between cell lines with HER2 overexpression and those without using the Mann–Whitney test for statistical comparison.

The effects of lapatinib on human endometrial cancer cells were evaluated using a panel of 19 established endometrial cancer cell lines that expresses widely varying levels of EGFR and HER2 (Table 1). These cell lines were selected to be representative of a range of endometrial cancer subtypes. Five cell lines (USPC1, USPC2, HEC155, SPAC1L, and SPAC1S) were obtained from patients with type II uterine serous papillary endometrial cancer (Hirai *et al*, 1994; Santin *et al*, 2002; Iida *et al*, 2004). The remaining cell lines are derived from type I endometrioid endometrial cancers. An exception is the cell line MFE319, which originates from an endometrial adenosquamous carcinoma (Hackenberg *et al*, 1997). To determine the relative effect of expression levels of HER2 and EGFR on lapatinib response, the EGFR and HER2 receptor content of the various cell lines used in the *in vitro* studies was quantitated using ELISA (Table 1). The receptor expression was further confirmed by western blot analysis (data not shown). Two out of the 19 established endometrial cancer cell lines (USPC1 and USPC2) demonstrated high levels of HER2 expression similar to the levels seen in the HER2-overexpressing breast cancer cell line SK-BR-3 known to have amplification of the HER2 gene. The HER2 protein levels in these HER2-overexpressing endometrial cancer cell lines ranged between 288 ng per mg protein (USPC1) and 1377 ng per mg protein (USPC2). Using FISH, both USPC lines demonstrated obvious HER2 gene amplification with USPC1 demonstrating an average of 10 signals per cell and USPC2 showing the presence of signal cluster(s) of more than 25 scattered signals per cell. Precise signal enumeration of high-level amplification was not possible because of coalescing fluorescence of signal cluster(s) (Figure 1B). Neither of the two HER2-amplified cell lines demonstrated coamplification of the topo-II-alpha gene (Figure 1B). Four of the established endometrial cancer cell lines (HEC155, SNG-II, SNG-M, and RL-95-2) expressed EGFR at high levels comparable to those seen in breast cancer cells known to express high level of EGFR (Konecny *et al*, 2006). The EGFR protein expression in these cell lines ranged between 98 ng per mg protein (HEC155) and 228 ng per mg protein (RL-95-2). All of the remaining established human endometrial cancer cell lines expressed varying but lower levels of EGFR compared with the HEC155, SNG-II, SNG-M, or RL-95-2 cells (Table 1).

After quantitation of EGFR and HER2 receptor levels in the endometrial cancer cell lines, growth assays were performed (Table 1 and Figure 1A). The effective dose range for each drug (IC_10_–IC_80_) was identified using a wide range of lapatinib concentrations (0.031–10 *μ*M). Lapatinib inhibited the proliferation of all endometrial cancer cell lines investigated in a concentration-dependent fashion; however, the IC_50_ values varied significantly between individual cell lines with up to a 200-fold difference in the IC_50_ values and ranged between 0.052 *μ*M in HER2-overexpressing USPC2 endometrial cancer cells and 10.9 *μ*M in MFE296 cells that express low levels of HER2 and EGFR (Table 1). Both HER2-overexpressing endometrial cancer cell lines had significantly lower IC_50_ values compared with nonoverexpressing cell lines (Figure 1C; mean IC_50_ value 0.33 *vs* 4.15; *P*=0.024), and EGFR expression was significantly inversely correlated with IC_50_ values (Figure 1C; Spearman *ρ*, *r*=−0.57, *P*=0.011). We next investigated the sensitivity of the endometrial cancer cell lines to lapatinib by using a binary classification, and thus two classes of cell lines were defined: sensitive and resistant. Using the median IC_50_ value as a cutoff cell lines with an IC_50_ below 3 *μ*mol were classified as sensitive and those with higher IC_50_ values as resistant. Using this binary classification, 4/5 uterine serous papillary cell lines and all 6 cell lines with either high HER2 (USPC1 and USPC2) or EGFR (HEC155, RL-95-2, SNG-II, and SNG-M) expression were classified as sensitive. In contrast, none of the resistant cell lines demonstrated HER2 overexpression or high levels of EGFR expression. Among those cell lines with low levels of HER2 and EGFR expression, only EFE184 demonstrated responsiveness to lapatinib at nanomolar concentrations (IC_50_ 0.649 *μ*M).

### Effects of lapatinib on HER2, EGFR, AKT, and ERK signalling in endometrial cancer cells

**Figure 2:**
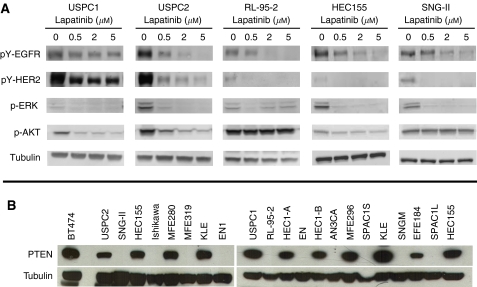
(**A**) Dose-dependent activity of lapatinib on HER2, EGFR, AKT, and ERK phosphorylation in USPC1, USPC2, RL-95-2, HEC155, and SNG-II cells. Both cell lines were treated with increasing doses of lapatinib (0.5–5 *μ*M) for 1 h. Immunoprecipitation and western blotting to detect phosphorylated HER2 or EGFR were performed as described in Materials and Methods. ERK and AKT phosphorylation levels were detected using phospho-specific ERK and AKT antibodies as described in Materials and Methods. (**B**) Western blotting to detect PTEN expression was performed as described in Materials and Methods.

As a measure of functional activity of the receptor tyrosine kinases, the phosphorylation state of HER2 or EGFR was assessed using immunoblotting techniques. Exposure of USPC1, USPC2, RL-95-2, HEC155, and SNG-II human endometrial cancer cells to lapatinib resulted in a dose-dependent reduction of phosphorylation of both the EGFR and HER2 receptor tyrosine kinases and their downstream signalling intermediates AKT and ERK (Figure 2A). Of note, lapatinib was not able to inhibit AKT phosphorylation in both RL-95-2 and SNG-II cells. RL-95-2 cells are known to be PTEN mutated (Gagnon *et al*, 2003). Inactivation of PTEN leads to activation of downstream PI3K with subsequent activation of the AKT survival pathway. A recent report suggests that inactivation of PTEN with failure to inhibit AKT leads to *in vitro* resistance towards EGFR TKIs (She *et al*, 2003). In our studies, however, both RL-95-2 and SNG-II were among the most sensitive cell lines despite lack of AKT inhibition through lapatinib. To better understand the association between PTEN deficiency and response to TKIs, we assessed PTEN expression in all of the 19 endometrial cancer cell lines using western blot analysis and compared the *in vitro* sensitivity towards lapatinib between lines with low and those with high expression levels (Figure 2B). Low expression of PTEN (as seen in SNG-II, Ishikawa, MFE319, EN1, RL-95-2, EN, AN3CA, SPAC1S, SNG-M, and SPAC1L cells) was not associated with resistance towards lapatinib when compared with those cell lines with high levels of PTEN expression (mean IC_50_ 4.09 *vs* 3.37, *P*=0.617).

### Novel predictive markers

**Figure 3:**
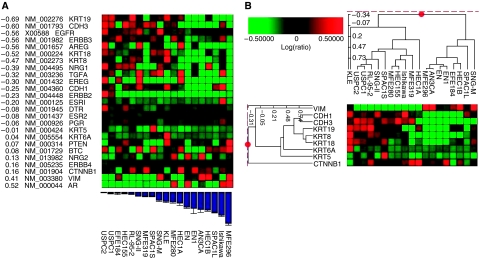
(**A**) The red and green matrices represent the normalised expression patterns for each gene in Table 2 across the 19 endometrial cancer cell lines. Brightest red indicates highest relative expression; brightest green indicates lowest relative expression. The reference cRNA pool consists of equal amounts of cRNA from the 18 endometrial cancer cell lines. Cell lines are ordered from low IC_50_ values to high IC_50_ values. Genes are weighted according to their correlation with the IC_50_ values. A negative correlation indicates that the marker is associated with sensitivity to lapatinib. (**B**) A cluster diagram of the 19 endometrial cancer cell lines was developed using the markers VIM, CDH1, CDH3, and the KRT5, 6, 8, 18, and 19. Post-EMT cell lines showed high expression of VIM, but low expression of CDH1, CDH3, and KRT8, 18, and 19 (eg, MFE296, HEC1B, AN3CA, EN1, EN, and EFE184).

A key aspect of therapies with targeted agents is accurate selection of patients most likely to benefit from therapy. Our preclinical data demonstrate that increased expression of either HER2 or EGFR was significantly associated with *in vitro* sensitivity to lapatinib (Table 1; Figure 1C). Moreover, we were able to demonstrate a significant inverse correlation between IC_50_ values and the relative expression of the autocrine EGFR ligand amphiregulin (*r*=−0.56, *P*=0.012), and HER3, the preferred oligomerisation partner of HER2 (*r*=−0.56, *P*=0.012), suggesting that lapatinib-sensitive cell lines have higher levels of amphiregulin or HER3 expression (Table 2; Figure 3A). Other EGFR ligands such as neuregulin 1, TGF-*α*, and epiregulin showed a similar inverse correlation with the IC_50_ values, although not at a statistically significant level (Table 2). We found no association between low ER-*α*, ER-*β*, or PR expression and improved *in vitro* response; however, lapatinib-sensitive cell lines did have lower levels of AR expression (*r*=0.52, *P*=0.021).

Induction of regulators of an EMT, such as Twist, Slug, and Snail, represses epithelial cadherin transcription in cancer cells, causing downregulation of this adhesive epithelial marker (Cowin *et al*, 2005). Conversely, mesenchymal markers, such as vimentin (VIM), are induced in these cancer cells, all leading to a less adhesive, more motile cell morphology that allows local tumour cell evasion (Cowin *et al*, 2005). A cluster diagram of the 19 endometrial cancer cell lines was developed using the markers VIM, E-cadherin (CDH1), P-cadherin (CDH3), and the cytokeratins (KRT) 5, 6, 8, 18, and 19 (Figure 3B). Several endometrial cancer cell lines were classified as representing endometrial cancer cells that have undergone an EMT (post-EMT). Post-EMT cell lines with high expression of VIM, and low expression of CDH1 or CDH3 also demonstrated lower expression levels of epithelial KRT such as 8, 18, and 19 (eg, MFE296, HEC1B, AN3CA, EN1, EN, and EFE184) (Figure 3B). These cell lines with post-EMT features tended to be less sensitive towards lapatinib when compared with those lines with an epithelial phenotype (mean IC_50_ 5.84 *vs* 2.78, *P*=0.079). Conversely, we were able to demonstrate inverse correlations between IC_50_ values and the relative expression of CDH1 (*r*=−0.25, *P*=0.283), CDH3 (*r*=−0.60, *P*=0.007), and the KRT8 (*r*=−0.47, *P*=0.043), 18 (*r*=−0.52, *P*=0.021), or 19 (*r*=−0.69, *P*=0.001), suggesting that the epithelial phenotype may be associated with lapatinib sensitivity.

### Combinations of lapatinib and chemotherapeutics

**Figure 4:**
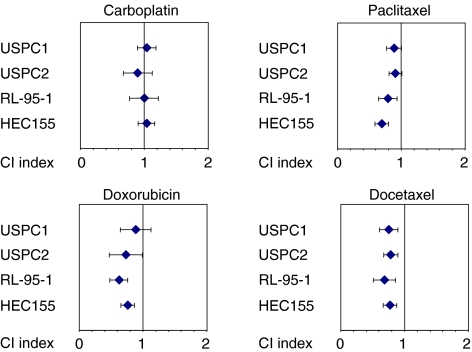
Mean CI values for chemotherapeutic drug–trastuzumab combinations in four different human endometrial cancer cell lines. Error bars indicate the 95% confidence interval of the mean value. Mean is derived from three replicates spanning clinically relevant concentration ranges sufficient to inhibit growth of control cells by 20–90%. Combination index values were derived from parameters of the median effect plots, and statistical tests were used to determine whether the CI values at multiple effect levels (IC_20_–IC_90_) were statistically significantly different from CI values equal to 1. Values that are statistically significantly less than 1 indicate synergistic interactions. Values that are statistically significantly greater than 1 indicate antagonistic interactions. Values equal to (or not statistically significantly different from) 1 indicate additive interactions.

In HER2-overexpressing breast cancer, the effect of chemotherapy can be maximised when combined with trastuzumab or lapatinib (Romond *et al*, 2005; Geyer *et al*, 2006). Thus, we next analysed the combination of lapatinib and chemotherapeutic agents used for the treatment of endometrial cancer in lapatinib-responsive endometrial cancer cell lines. Multiple drug effect analysis was performed using two HER2-overexpressing and two EGFR-expressing established endometrial cancer cell lines to determine the nature of the interaction between lapatinib and carboplatin, paclitaxel, doxorubicin, or docetaxel (synergy, addition, or antagonism). The lapatinib concentrations used for these experiments ranged between 0.031 and 1.0 *μ*M for USPC1 and USPC2 cells, and 0.31 and 10.0 *μ*M for RL-95-2 and HEC155 cells and were below the reported peak plasma concentrations achievable in humans (Burris *et al*, 2004). The drug concentrations of carboplatin, paclitaxel, doxorubicin, and docetaxel used for these experiments have been published previously (Pegram *et al*, 2004). Additive interactions were observed in all four endometrial cancer cell lines for lapatinib plus carboplatin (mean CI values ranged from 0.90 (*P*=0.358) to 1.04 (*P*=0.564)) (Figure 4). Synergistic and additive interactions were observed for lapatinib plus paclitaxel (0.69 (*P*<0.001) to 0.91 (*P*=0.068)) and doxorubicin (0.62 (*P*<0.001) to 0.89 (*P*=0.341)) (Figure 4). Synergistic interactions were observed for lapatinib plus docetaxel (0.68 (*P*=0.001) to 0.78 (*P*<0.001)) in all four cell lines (Figure 4).

## DISCUSSION

Evidence for a role of HER2 and EGFR in the pathogenesis of various cancers has led to the rational design and development of agents that selectively target HER2 and EGFR. In unselected patients with endometrial cancer, HER2 gene amplification/overexpression represents a rare event. However, HER2 amplification/overexpression is seen more commonly in well-defined subtypes of endometrial cancer such as uterine serous papillary cancer, clear cell cancer, or grade 3 endometrioid cancer (Morrison *et al*, 2006). Trastuzumab (Herceptin™) a humanised anti-HER2 antibody has recently been approved for the adjuvant treatment of HER2-overexpressing (3+ IHC) or FISH-positive primary breast cancers based on a highly significant reduction in the risk of recurrence of 52% in node-positive HER2-positive primary breast cancer (Romond *et al*, 2005). Encouraged by these data, we intended to evaluate the therapeutic potential of a dual kinase inhibitor targeting both HER2 and EGFR for the treatment of endometrial cancer and proposed to define markers that may help identify patient subsets that would preferentially benefit from such a target-based therapy.

To date, no studies have been reported that investigate the activity of lapatinib in endometrial cancer cells. Here, we show concentration-dependent anti-proliferative effects of lapatinib in all endometrial cancer cell lines tested. The response, however, varied significantly between individual cell lines with up to a 200-fold difference in the IC_50_ values. As expected, significantly lower IC_50_ values were associated with HER2 amplification/overexpression. Surprisingly, increased EGFR expression was also associated with improved *in vitro* response to lapatinib. This is remarkable as it is in contrast to preclinical studies in breast cancer, where no significant association between EGFR expression and *in vitro* sensitivity to lapatinib was seen across a large panel of breast cancer cell lines (Konecny *et al*, 2006). Epidermal growth factor receptor may play a different role in endometrial cancer compared with breast cancer. Importantly, the clinical benefit observed with anti-EGFR TKIs across different disease entities has been variable (eg, EGFR TKIs are largely inactive in colorectal cancer); however, two of these drugs, gefitinib and erlotinib, have demonstrated clinical activity in nonsmall cell lung cancer (NSCLC) and responses have been observed in patients with advanced pancreatic cancer and in head-and-neck cancer (Baselga, 2006). The current preclinical data suggest that EGFR inhibitors may also be clinically active in well-defined subgroups of endometrial cancer. Earlier clinical studies in other disease entities show that potential markers of sensitivity to EGFR TKIs include the presence of EGFR gene amplification, mutations of the EGFR gene, and increased expression of EGFR ligands (Baselga and Arteaga, 2005). The role of EGFR gene amplification or mutations in endometrial cancer has not been studied yet. Our preclinical findings, however, do suggest that increased expression of EGFR ligands such as amphiregulin, TGF-*α*, epiregulin, or NRG1 may also be associated with sensitivity to lapatinib in endometrial cancer.

HER3 is the preferred heterodimerisation partner of HER2. Ligand-induced stimulation of HER3 leads to the activation of HER2 by transduction through heterodimerisation. Preclinical data suggest that HER3 overexpression increases sensitivity to TKIs such that sensitivity towards gefitinib was only seen in NSCLC cell lines with increased expression of HER3 (Engelman *et al*, 2005). Similarly, we also found a positive association between HER3 expression levels and increased *in vitro* sensitivity to lapatinib using the current panel of endometrial cancer cell lines. Higher levels of HER3 expression may promote hetero-oligomer formation with HER2 and EGFR and lead to increased activation of the PI3K/AKT pathway. This pathway activation may in return lead to increased sensitivity towards EGFR or HER2 inhibition by lapatinib. Nevertheless, further research will be necessary to clearly define the clinical value of HER3 as a predictive marker for small molecule receptor TKIs in endometrial cancer.

An inverse association has been described between HER2 amplification/overexpression and the presence of receptors for the steroid hormones oestrogen and progesterone in both clinical correlative studies and experimental models for both breast and endometrial cancer (Bigsby *et al*, 1992; Konecny *et al*, 2003; Mariani *et al*, 2005). Preclinical data demonstrate that HER2 overexpression can lead to ligand-independent activation of the ER and a reduction in the oestrogen-binding capacity of the cells (ie, to decreased ER levels) and to a decrease in ER as well as PR transcripts (Pietras *et al*, 1995). Moreover, recent laboratory studies similarly confirm ligand-independent activation of the AR through HER2 in prostate cancer cells (Sugita *et al*, 2004). Consistent with these experimental findings, high EGFR and HER2 expression was associated with low AR expression in the present endometrial cancer cell line panel, thus suggesting that low AR expression could be a useful surrogate marker for receptor tyrosine kinase activation in endometrial cancer, possibly more useful than ER or PR may be. However, further research will be necessary to help define the predictive value of AR in endometrial cancer, which in a recent study was found to be expressed in 39 out of 44 (89%) patients with endometrioid endometrial cancer (Ito *et al*, 2002).

Accumulating evidence points to a critical role of EMT-like events during tumour progression. In this study, we found an association between *in vitro* resistance to lapatinib and a post-EMT cell line phenotype. Using the baseline expression arrays we were able to discriminate ‘less responsive’ and ‘more responsive’ cell lines using the differential expression of six genes: VIM, CDH1, CDH3, and KRT8, 18, or 19. While other genes may be derived from the analysis of large microarray data sets, the biologic relevance of these genes makes them plausible predictive markers worth further preclinical and clinical evaluation.

Antitumour activity of the HER2 antibody trastuzumab in HER2-overexpressing breast cancers seems to be dependent upon the presence of PTEN, a phosphatase that inhibits phosphatidylinositol 3-kinase-Akt signalling. Recently, it has been proposed that trastuzumab can activate PTEN, which contributes to the trastuzumab antitumour function (Nagata *et al*, 2004). Consequently, PTEN deficiency, which occurs in up to 50% of breast cancers, may predict for resistance to trastuzumab therapy (Nagata *et al*, 2004). In contrast, however, recent preclinical studies with lapatinib, demonstrate that the small molecule inhibitor exerts its antitumour activity in a PTEN-independent manner (Xia *et al*, 2007). Our results confirm these observations in that lapatinib activity was not associated with PTEN status in endometrial cancer cells. The antitumour activity of lapatinib in a PTEN-independent manner may be of particular importance for endometrial cancer as PTEN deficiency is the most common genetic defect in endometrioid carcinoma and is seen in up to 83% of tumours that are preceded by a histologically discrete premalignant phase (Hecht and Mutter, 2006).

Patients most likely to be candidates for systemic therapies are high-risk endometrial cancer subgroups with deep myometrial invasion, positive lymph nodes, or type II endometrial cancers with nonendometrioid histology (usually papillary serous or clear cell). These are the patients most likely to also benefit from adjuvant-targeted therapies. Our laboratory data provide a clear biologic rational to test lapatinib as a single agent or in combination with chemotherapy in HER2-overexpressing and/or possibly EGFR-expressing endometrial cancer. Patients most appropriate to screen for HER2 amplification are patients with nonendometrioid histologies (type II). Further evaluation of not only EGFR ligands and HER3 levels but also AR levels and markers of EMT may help to define patients with EGFR-expressing endometrial cancer most likely to respond to lapatinib.

## Figures and Tables

**Table 1:** Lapatinib concentrations that achieve 50% growth inhibition (IC_50_), and the corresponding levels of HER2 and EGFR expression as measured by ELISA

**Cell line**	**IC_50_ (*μ*mol)**	**SE**	**HER2 (ng mg^−1^)**	**SE**	**EGFR (ng mg^−1^)**	**SE**
USPC2	0.052	0.370	1377	39	66	9.0
USPC1	0.607	0.245	288	16	38	1.0
EFE184	0.649	0.436	1.9	0.5	9.0	0.4
HEC155	0.790	0.052	5.0	0.1	98	1.7
RL-95-1	0.927	0.077	3.5	1.0	228	18
SNG-II	1.534	0.153	8.1	0.6	208	6.8
MFE319	1.597	0.267	7.7	1.0	22	1
SPAC1S	1.652	0.274	10	1.3	24	4.0
SNG-M	3.26	0.529	13	0.3	103	3.4
KLE	3.298	0.420	17	3.0	23	4.0
MFE280	3.566	0.207	3.6	1	1.2	0.3
EN1	3.756	0.367	22	4	29	1.3
HEC1A	4.011	0.872	4.1	1	36	1.3
EN	5.334	0.450	14	0.9	2.7	0.9
AN3CA	6.099	1.187	3.0	1	12	1.3
HEC1B	6.368	0.424	8.8	1	9.4	1.2
SPAC1L	7.102	0.302	6.9	0.5	18	3.8
Ishikawa	8.137	0.503	14	1.5	15	3.3
MFE296	10.973	0.723	2.93	1.0	2.85	1.0

EGFR=epidermal growth factor receptor.

**Table 2:** Correlations between IC_50_ values and the relative expression of biomarkers in the 19 endometrial cancer cell lines

**Name**	**Symbol**	**Spearman's *ρ***	***P*-value**
EGFR	EGFR	**−0.56**	**0.013**
HER2	HER2	−0.23	0.336
HER3	HER3	**−0.56**	**0.012**
HER4	HER4	0.16	0.514
Epiregulin	EREG	−0.30	0.209
Amphiregulin	AREG	**−0.56**	**0.012**
Transforming growth factor-*α*	TGFA	−0.32	0.177
Heparin-binding EGF-like growth factor	DTR	−0.08	0.737
Betacellulin	BTC	0.08	0.742
Neuregulin 1	NRG1	−0.39	0.101
Neuregulin 2	NRG2	0.13	0.586
Estrogen receptor-*α*	ESR1	−0.20	0.403
Estrogen receptor-*β*	ESR2	−0.08	0.737
Progesterone receptor	PGR	−0.06	0.797
Androgen receptor	AR	**0.52**	**0.021**
E-Cadherin	CDH1	−0.25	0.283
P-Cadherin	CDH3	**−0.60**	**0.007**
*β*-Catenin	CTNNB1	0.16	0.495
Vimentin	VIM	0.41	0.078
Cytokeratin 5	KRT5	−0.01	0.988
Cytokeratin 6	KRT6	0.04	0.858
Cytokeratin 8	KRT8	**−0.47**	**0.043**
Cytokeratin 18	KRT18	**−0.52**	**0.021**
Cytokeratin 19	KRT19	**−0.69**	**0.001**

EGFR=epidermal growth factor receptor.

Significant correlations are given in bold.
